# Convolution neural network with batch normalization and inception-residual modules for Android malware classification

**DOI:** 10.1038/s41598-022-18402-6

**Published:** 2022-08-17

**Authors:** TianYue Liu, HongQi Zhang, HaiXia Long, Jinmei Shi, YuHua Yao

**Affiliations:** 1 College of Information Science Technology, Hainan Normal University, No.99 LongKun South Road, Haikou city, 571158 Hainan Province China; 2 College of Information Engineering, Hainan Vocational University of Science and Technology, No.18 QiongShan Road, Haikou city, 571126 Hainan Province China; 3 College of Mathematics and Statistics, HaiNan Normal University, No.99 LongKun South Road, Haikou city, 571158 Hainan Province China

**Keywords:** Computer science, Information technology

## Abstract

Deep learning technology is changing the landscape of cybersecurity research, especially the study of large amounts of data. With the rapid growth in the number of malware, developing of an efficient and reliable method for classifying malware has become one of the research priorities. In this paper, a new method, BIR-CNN, is proposed to classify of Android malware. It combines convolution neural network (CNN) with batch normalization and inception-residual (BIR) network modules by using 347-dim network traffic features. CNN combines inception-residual modules with a convolution layer that can enhance the learning ability of the model. Batch Normalization can speed up the training process and avoid over-fitting of the model. Finally, experiments are conducted on the publicly available network traffic dataset CICAndMal2017 and compared with three traditional machine learning algorithms and CNN. The accuracy of BIR-CNN is 99.73% in binary classification (2-classifier). Moreover, the BIR-CNN can classify malware by its category (4-classifier) and malicious family (35-classifier), with a classification accuracy of 99.53% and 94.38%, respectively. The experimental results show that the proposed model is an effective method for Android malware classification, especially in malware category and family classifier.

## Introduction

In the last decade, mobile communication has had an incredible impact on the society and economy with the gradual proliferation of smart phones and the rapid development of mobile networks^1^. Mobile networks have enabled significant changes in the way people learn, work, and live, with technological changes, scientific innovations, and the development of artificial intelligence making everything more convenient. Advanced mobile technologies have made mobile devices powerful and rich in features such as Internet browsing, payment, and photography. According to the Ericsson 2021 report^2^, mobile data traffic has grown 300 times since 2011, with 5.5 billion new mobile devices connected online worldwide. The huge data traffic necessitates more intelligent operating systems, and Android is the most widely used platform for mobile devices and IoT devices^3^.

Android, which is currently the most widely used mobile operating system, has been the main target of malware attacks. According to the latest report^4^, mobile data traffic grew by 46% from the first quarter of 2020 to the first quarter of 2021. By the end of 2021, there will be approximately 580 million 5G users worldwide, more than double the number of users at the end of 2020 (220 million), and by the end of 2026, it is expected to reach 3.5 billion. Such large amounts of traffic create considerable ease for malware to emerge, and as the number of malware increases, so do the variants of malware. Malware attacks on Android phones currently account for 72.2% of the total market share^5^. Hackers attempt to attack smart phones by using a variety of methods such as sending spam messages, threatening messages, and malicious advertisements. Faced with the endless attack methods of hackers and the spread of a large amount of malware, how to effectively detect malware on Android platforms and classify malware categories and families has become an important research topic in the field of cybersecurity. With the rapid development of deep learning (DL) technology, the application of DL methods for the detection and classification of Android malware has become a research direction of interest.

To address this issue, in this paper, we present a novel approach for the classification of Android malware based on the improved convolution neural network (CNN). Concretely, we analyze the data traffic packets circulated by the application and perform supervised malware classification by parameterizing the characteristics of these packets. Our contributions are listed as follows: 1) A new feature extraction method, 347-dim network traffic features is proposed through aggregating the features vectors. 2) Convolution neural network is combined with batch normalization and inception-residual network modules (BIR-CNN) which help to improve network performance, convergence rate and over-fitting. 3) The accuracy of the deep learning model BIR-CNN exceeds some state-of-the-art methods with 99.73% in binary classification (2-classifier) and 99.53% in category (4-classifier) and 94.38% in malicious family (35-classifier) respectively, especially, the results of multi classification are greatly improved.

## Related work

Traditional malware detection methods include static detection techniques, dynamic detection techniques, and hybrid detection techniques^6^. Static detection techniques involve systematic detection and analysis based on application features without running the APP^7–10^. Generally, unpacking or disassembling is performed to detect the features of the APP. Commonly used features include application permissions, strings, and network addresses. However, static detection techniques cannot effectively identify certain source code modification behaviors. In contrast, dynamic detection methods can detect the execution behavior of an application or track tainted behavior such as system calls, network connections, and memory utilization^11–13^. However, dynamic detection techniques can detect malware only at runtime and cannot trace the execution path or detect certain malicious behaviors at any time. To overcome the limitations of static and dynamic detection analysis methods, researchers have used a combination of both mechanisms, known as hybrid analysis detection methods^14,15^. It is a two-step process that first utilizes static analysis and then uses dynamic analysis methods. It is evident from the aforementioned analysis methods that researchers rarely consider the network traffic of such malware.

Currently, almost all attackers use mobile networks to obtain users’ no-dare information or to carry out with malware; thus, the detection of Android malware by the analysis of network traffic becomes possible. Researchers have analyzed network traffic for Android malware detection, and now the technology of using network traffic to detect malware has become one of the key points in network security research. In 2016, Murtaz et al.^16^ proposed a method for detecting whether an application is malicious by performing only nine network traffic metrics; moreover, they compared this method with models such as random forest (RF), k-nearest neighbor (KNN), decision tree (DT), random tree (RT), and logistic regression (LR), and finally obtained a detection accuracy of 94%, which is higher than that of other malware detection methods; however, there is still room for improvement. In the same year, Amrute et al.^17^ proposed an Android malware detection method based on network traffic and combined it with the Logcat and Dalvik Debug Monitor Server (DDMS ) techniques. In 2018, Zulkifli et al.^18^ proposed a dynamic detection technique based on network traffic; they extracted features from the Drebin and CataGuiDupppSet dataset and inputted them into a J48 DT model, and obtained an accuracy of 98.4% in the Drebin dataset. In 2019, some researchers processed the network traffic and used it as a feature for Android malware detection and classification. For example, Li et al.^19^ proposed a detection model based on machine learning (ML), extracted network traffic features with robustness, and trained a detection model that can identify unknown mobile malicious network traffic. They achieved a detection accuracy of 90% for unknown malicious samples and an F1-score of 80%. Although the model is relatively novel, its detection accuracy needs to be improved. Wang et al.^20^ proposed a method for extracting the temporal feature relationships in network traffic, and they detected malware by using a two-layer bidirectional long short-term memory (LSTM) recurrent neural network model; however, the experimental results were not satisfactory. Chen et al.^21^ employed highly unbalanced network traffic as features and used support vector machine (SVM) and improved SVM cost-sensitive (SVMCS ) model to detect malware.

Network traffic can be used for the detection and classification of malware. In 2018, Lashkari et al.^22^ collected approximately 6000 benign applications as well as malware from Google Play and other sources and let these applications run in a real smart phone environment to obtain a new Android malware dataset. This dataset incorporates the shortcomings and limitations of all previous dataset^23^. They inputted 80 traffic features into RF, KNN, and DT models to detect and classify malware, achieving an average accuracy of 85% and a recall of 88%, while the accuracy of classification was only 44.3%. In 2020, Abuthawabeh et al. proposed a supervised model named extra-trees^24^ based on conversation-level network traffic features for Android malware detection, classification, and family categorization. The proposed method achieves the highest weighted accuracy of 87.75%, 79.97%, and 66.71% in malware detection, malware classification, and malware family classification, respectively. Compared with the method proposed by Lashkari et al.^22^, it achieves a significant improvement in the accuracy of malware family classification but a relative decrease in the accuracy of malware detection and malware category classification.

The above works entailed the detection and classification of malware by using traditional ML methods by using network traffic as a feature. Although the accuracy of malware detection is high, the effectiveness of malware classification needs to be improved.

The application of DL for malware detection and classification was proposed as early as 2018. Sabhadiya et al.^25^ developed used a combination of static and dynamic features fed into recurrent neural networks , which is much more efficient than logistic regression and SVM. In 2019, Taheri et al.^26^ performed malware detection and classification based on the CICAndMal2017 dataset. They developed a two-layer malware classifier by using permissions and intent as static features and API calls as dynamic features and achieved a detection accuracy of 95.3%, a malware category classification accuracy of 83.3%, and a malware family classification accuracy of 59.7% in the second layer.

In 2020, Guo et al.^27^ proposed a CNN model-based application traffic classification algorithm for addressing the problems of large computation and low efficiency encountered in traditional ML algorithms during traffic feature extraction. Its classification accuracy and recall were 94.28% and 95.15%, respectively. However, they used the algorithm only for supervised traffic data and did not address the problem of unlabeled data. Feng et al.^28^ proposed a two-layer classifier, where the second layer uses network traffic as features combined with fully connected neural networks for detection; the overall detection accuracy was 99.3%: 98.2% for malware categories and 71.48% for malicious families. Compared with traditional malware detection and classification methods, this method exhibits a substantial improvement in the accuracy of community detection. In 2021, Zhou et al.^29^ directly read the bit group of classes.dex as the base data, processed it as the pixel value of an image, and then used a CNN model for detection, which is innovative; however, data processing as an image entails strict equipment requirements. In the second half of 2021, Gohari et al.^30^ directly extracted local features from network traffic by using a one-dimensional CNN and then used LSTM to detect the order between features; they obtained a malware detection accuracy of 99.79%, a malware category classification accuracy of 98.90%, and a malware family classification accuracy of 97.29%. Compared with the previous literature, they obtained considerably higher malware classification accuracy.

The aforementioned studies involve many methods for classification of malicious traffic; some articles directly combined network traffic as features and CNN for malware detection and classification. However, our approach differs from the above mentioned methods in several aspects. First, we propose a novel CNN model that combines network traffic features by using five clustering algorithms, namely mean, median, standard deviation, skew, and kurtosis. Second, this model combines inception-residual network modules on top of the traditional CNN and can thus identify Android malware with high accuracy and classify them by category and malicious family. In the following sections, we will discuss our model and network traffic extraction method in more detail and present the operational procedure of our method.

## Methods

**Figure 1:**
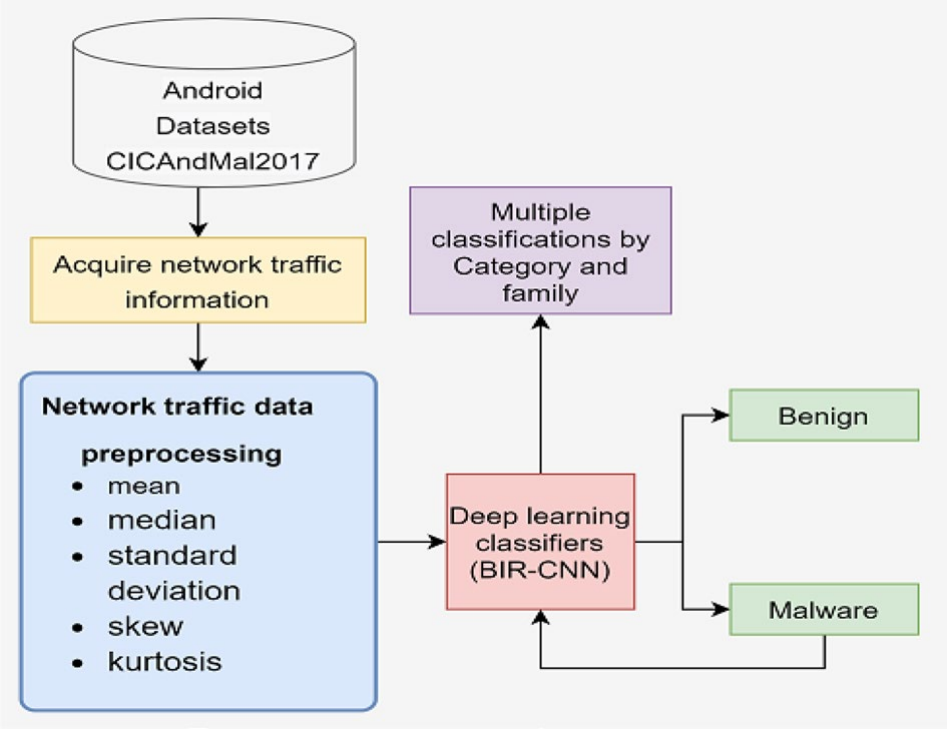
Overall architecture of the proposed model.

In this section, we propose a new model for classifying malware. First, a new dataset aggregation is used to extract network traffic features and obtain 347-dim features; then, the model developed in this study, BIR-CNN deep learning model, is used to detect whether the dataset is benign applications or malware, and classify malware by category and family. The complete framework is illustrated in Fig. 1.

### Dataset feature extraction

**Figure 2:**
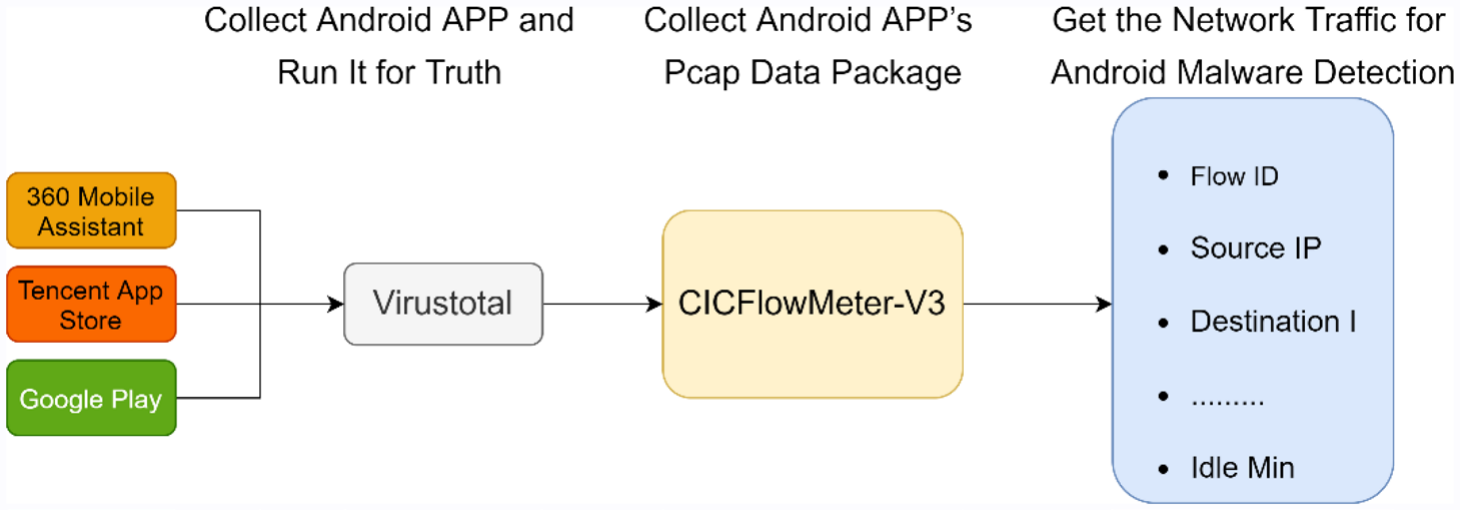
Data Extraction Process.

To classify Android malware, we use the CICAndMal2017 dataset^22^, which collects all network traffic generated during the execution of an application within a given time interval after installation. The CICAndMal2017 dataset collected 4354 malware and 6500 benign apps from VirusTotal, Contagio security blog, and previous studies. Because of sample errors and repeated labels in different dataset, CICAndMal2017 finally retained 429 as malware and 5065 as benign. Network traffic data are captured in three stages. Because advanced malware mostly uses evasion or conversion techniques such as code swapping, register renaming, and idle activation to avoid detection, some malicious application behaviors are triggered only after connecting to the network for updates, and certain malware behaviors are only triggered during reboots. To trigger all the malware behaviors, network traffic data is captured 3 min after an app is installed and 15 min before and after restarting the phone. Figure 2 shows the data extraction process.

The dataset consists of 2126 samples, where each sample corresponds to one instance of an android application installed and executed on a mobile phone. For each sample, all network flows appearing in the network during execution are captured. For each flow, 84 features are recorded. Each sample has three different labels: (1) a binary label indicating whether the application is malicious, (2) a category label with four possible values indicating the general class of malware, and (3) a family label with 35 different values indicating the specific type of malware. Some of the types, along with their associated malware category, are listed in Table 1.

**Table 1:** Malware category and family types.

Category	Family tree		
Benign	Benign 2015	Benign 2016	Benign 2017
Adware	Dowgin	Ewind	Feiwo
	Gooligan	Kemoge	Koodous
	Mobidash	Shuanet	Youmi
Ransomware	Charger	Jisut	Koler
	Lockerpin	Pletor	PornDroid
	RansomBO	Simplocker	SVpeng
	WannaLocker		
Scareware	AndroidDefender	AVforAndroid	AVpass
	FakeApp	FakeAppAL	FakeAV
	Penetho	VirusShield	
SMSmalware	Biige	Fakeinst	FakeMart
	FakeNotify	Jifake	Nandrobox
	Plankton	Zsone	

In this paper, the dataset was processed as follows. First, the data with less than nine samples belonging to a particular malicious family were removed to ensure a reasonable split into training, validation, and test sets. After this operation, only 2071 samples were left. A feature set can be extracted for each sample. Second, the flow-id, timestamp, and endpoint IP and port information were removed from the feature set. Then, the vectors of the remaining features of all flows were obtained using the mean, median, standard deviation, skew, and kurtosis functions, and the aggregate values were concatenated, resulting in a 5-dim feature vector for each feature. There were 80 vectors for each sample; thus, 400-dim feature vectors were obtained for each sample. Finally, the features with all zeros and all constant feature columns were removed, leaving 347-dim feature vectors for each sample; moreover, all feature matrices were standardized before training.

### BIR-CNN model

**Figure 3:**
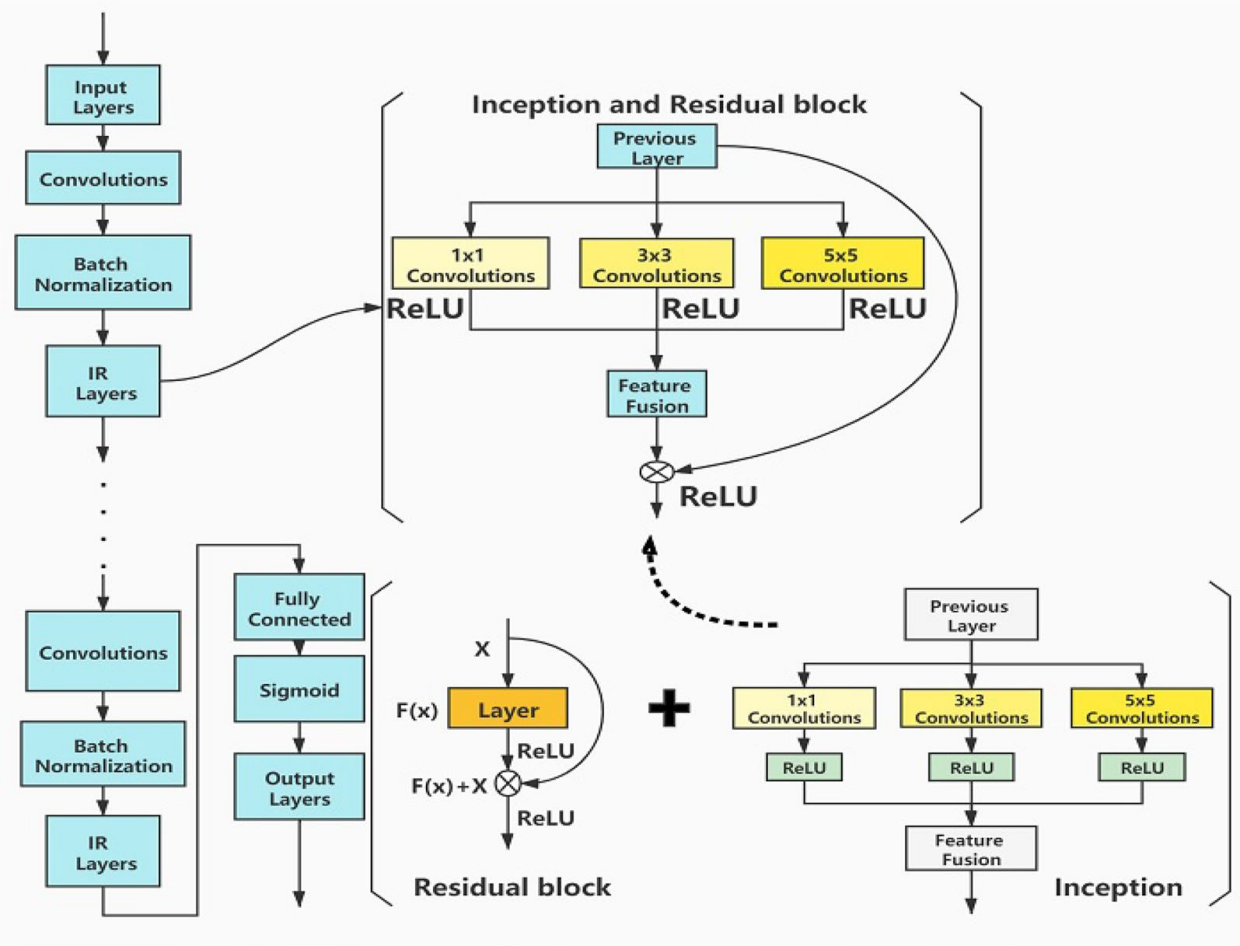
BIR-CNN model for malicious software detection and classification.

In this paper, we present a method to detect and classify Android malware by using a CNN based on batch normalization and inception-residual network. The structure of the model is illustrated in Fig. 3.

The CNN^31^ is a class of feed-forward neural network with a deep structure that includes end-to-end convolution computation for feature extraction and is one of the representative algorithms of DL. It has representation learning capabilities and can translate shift-invariant classification of input information according to its hierarchical structure. In the feed-forward neural network, the calculation process is as follows:

1 $$y = f\left( {\mathop \sum \limits_{i = 1}^{M} w_{i} x_{i} + w_{0} } \right)$$

where $w_{i}$ are the weight coefficients of the neural network, $w_{0}$ is the offset of the neural network, and $f$ is a nonlinear function that can be applied to a complex model.

The CNN model mainly includes the following layers: input layers, convolution layers, fully connected layers, and output layers. The convolution layer is mainly used for feature extraction and contains several convolution kernels. Each element of the convolution kernel corresponds to a weight coefficient and a deviation amount, and the size of the convolution kernel is called the field of view of the convolution layer. The result of CNN model training is closely related to the setting of the convolution layer. The output of the convolution layers can be calculated as follows:

2 $$J_{u,v}^{\left( l \right)} = \mathop \sum \limits_{i = - \infty }^{\infty } \mathop \sum \limits_{j = - \infty }^{\infty } x_{i + u,j + v}^{{\left( {l - 1} \right)}} k_{{rot_{i,j} }}^{\left( l \right)} \cdot X\left( {i,j} \right) + b^{\left( l \right)}$$

where $l-1$ is the input layer, ${X}_{m\times m}^{\left(L-1\right)}$ is the input feature map, ${K}_{\left(n\times n\right)}^{\left(l\right)}$ is the convolution kernel corresponding to the feature, and ${b}^{\left(l\right)}$ is the offset added to the output.

To alleviate the problem of gradient dispersion and speed up the convergence of the model, batch normalization is used in this paper to make the training of the deep network model easier and more stable. The function of batch normalization can be expressed as follows:

3 $$y = \frac{\gamma }{{\sqrt {Var\left[ x \right] + \epsilon } }} \cdot x + \left( {\beta - \frac{\gamma E\left[ x \right]}{{\sqrt {Var\left[ x \right] + \epsilon} }}} \right)$$

where $Var [x]$ is the variance under batches, $E [x]$ is the mean, and $\gamma$ and $\beta$ are the learning parameters.

The inception network modules is added in the convolution layer to improve the network performance by increasing the depth of the network. In each inception module, convolution kernels of different sizes are used, which can be interpreted as different receptive fields; then, they are concatenated, enriching the information in each layer. The receptive field can be calculated as follows:

4 $$RF_{i} = \left( {RF_{i + 1} - 1} \right) \times S_{i} + K_{i}$$

where $S$ is the step size of the convolution and $K$ is the size of the convolution kernel in this layer.

As the depth of the network increases, the training effect of the network decreases; this phenomenon is called network degradation. To construct a network with a large depth and to enhance the learning ability of the model, a residual network is employed in this paper because it does not experience network degradation as the depth of the network increases and the network performance becomes optimum. Residual block is used for all convolution layers; a building block is defined as

5 $$y = F\left( x \right) + x$$

where $x$ and $y$ are respectively the input and output vectors of the layers considered, and $F\left(x\right)$ is the function of residual mapping to be learned.

The choice of different perceptual fields in the convolution layer affects the final training effect of the model; therefore, the appropriate perceptual field must be chosen. Convolution kernels of different sizes are used to obtain different sizes of perceptual fields. Finally, different scales are spliced to achieve feature fusion, which helps obtain richer features. The network can choose the preferred perceptual field independently according to the learning. In the experiment, three sizes, namely 1 × 1, 3 × 3, and 5 × 5, were selected.

In our model, the two-point cross-entropy loss function is employed; its formula is as follows:

6 $$Loss = \frac{1}{N}\mathop \sum \limits_{i} - \left[ {\alpha_{i} \cdot \log p_{i} + \left( {1 - \alpha_{i} } \right)\log \left( {1 - p_{i} } \right)} \right]$$

where ${\alpha }_{i}$ is the confidence that the model predicts whether it is malicious code, and ${p}_{i}$ is the sample probability of predicting a positive class. If the sample is positive, then $\alpha_{i} = 1$; otherwise $\alpha_{i} = {0}$ .

Furthermore, the Sigmoid function is used in the paper to receive the value outputted by the output layer and then transform the value between 0 and 1, thus facilitating the classification operation and applying the dichotomous cross-entropy loss function. The formula for Sigmoid function is as follows:

7 $$Sigmoid\left( x \right) = \frac{1}{{1 + e^{ - x} }}$$

Gradient activation function (GAF)^32^ is used as the activation function in the BIR-CNN model. GAF is one of the lasted activation functions in artificial neural networks. Compared with traditional activation functions, GAF can deal with the ill-conditioned problem, the vanishing/exploding gradient problem, and the saddle point problem of deep neural networks. GAF enlarges the tiny gradients and restricts the large gradient. The formula for GAF is as follows:

8 $$GAF = \, \alpha (ln\left( {ReLU\left( {\beta g} \right) \, + \, 1} \right) - ln(ReLU( - \beta g) \, + \, 1))$$

where *α* and* β* are factors that control the shape of the GAF, *g* is a gradient vector, ReLU is rectifier linear unit.

The model proposed in this paper has convolution and fully connected layers in addition to dropout layers. The dropout layer allows the activation value of a neuron to stop working with a certain probability so that the network does not rely too much on some specific features and thus makes the entire network less complex structurally, alleviating over-fitting and making the model more generalizable.

### Evaluation metrics

The BIR-CNN is an ML model, and detection and classification of malicious software constitutes a two-classification problem. In this paper, we use the following evaluation indicators to evaluate the performance of the ML model.

#### True-positive (TP)

Positive samples are predicted by the model as positive classes. False-positive (FP): Negative samples are predicted by the model as negative classes. True-negative (TN): Negative samples are predicted by the model as positive classes. False-negative (FN): Positive samples are predicted by the model as negative classes.

#### Accuracy

For a given test dataset, the proportion of the number of samples correctly classified by the model to sample size of all. The formula is shown as follows:

9 $$Accuracy = \frac{TP + TN}{{TP + FN + TN + FP}}$$

#### Precision

It refers to the proportion of truly correct samples among the samples whose prediction results are positive. The formula is expressed as follows:

10 $$Precison = \frac{TP}{{TP + FP}}$$

#### Recall

It refers to the proportion of samples that are predicted to be positive in the total positive samples. The calculation formula is:

11 $${\text{Re}} call = \frac{TP}{{TP + FN}}$$

#### F1-score

It is a combination of precision and recall. Precision and recall are mutually exclusive: when one increases, the other decreases accordingly. To reconcile the two indicators, F1-Score is introduced:

12 $$F1 = 2 \times \frac{Precision \times Recall}{{Precision + Recall}}$$

#### Kappa coefficient

It is a metric used to test the consistency and can also measure the effectiveness of the classification. Therefore, for the classification problem, this coefficient can test whether the model prediction results are consistent with the actual classification results. Kappa coefficient is calculated based on the confusion matrix, which takes values between -1 and 1, usually greater than 0. The calculation formula is

13 $$kappa = \frac{{p_{0} - p_{e} }}{{1 - p_{e} }}$$

where ${p}_{0}$ denotes the overall classification accuracy and ${p}_{e}$ refers to the chance consistency error.

#### Receiver operating characteristic (ROC) curve

It is also known as the receptivity curve. Points on the curve reflect the response to the same signal stimulus, but the result is obtained under several different judgment criteria. The curve is obtained by connecting the points with the False Positive Rate (FPR) as the X coordinate and the True Positive Rate (TPR) as the Y coordinate.

14 $$FPR = {\raise0.7ex\hbox{${FP}$} \!\mathord{\left/ {\vphantom {{FP} {FP + TN}}}\right.\kern-\nulldelimiterspace} \!\lower0.7ex\hbox{${FP + TN}$}}$$

15 $$TPR = {\raise0.7ex\hbox{${TP}$} \!\mathord{\left/ {\vphantom {{TP} {TP + FN}}}\right.\kern-\nulldelimiterspace} \!\lower0.7ex\hbox{${TP + FN}$}}$$

#### Area under curve (AUC)

It is defined as the area under the ROC curve and is an important indicator of the classifier’s merit. When its value is closer to 1, the authenticity of the detection method is higher; when it is equal to 0.5, its authenticity is the lowest and has no application value. The calculation formula is

16 $$AUC = \sum\nolimits_{{i\epsilon positiveClass}} {\frac{{rank_{i} - \frac{{M\left( {1 + M} \right)}}{2}}}{{M \times N}}}$$

where *M* is the number of positive class samples, *N* is the number of negative class samples, and ${rank}_{i}$ refers to positive sample score greater than negative samples.

#### Precision recall (PR) curve

It is the line formed by connecting the points with the Recall as the X coordinate and the Precision as the Y coordinate. It is used to evaluate the classification performance of ML algorithms for a given dataset and corresponds spatially to ROC curves when the recall is not equal to 0, and the confusion matrix between the two coincides.

## Results

In this section, we first provide a detailed description of the dataset and the experimental environment. Then we provide the 2-, 4-, 35-classifier results of 347-dim network traffic features and BIR-CNN compared with other feature extraction methods and ML models. At last, we provide the 2-, 4-, 35-classifier results of BIR-CNN model which is compared with other ML models based on our proposed network traffic features.

### Experimental environment

The running and testing environment of the BIR-CNN model is Intel (R) i7-11,700 CPU, 32 GB memory, GeForce RTX™ 3090 Ti GPU, based on Windows10 operating system. The experiments are conducted on the CICAndMal2017 dataset^22^. The steps for processing each sample have been described in Section dataset feature extraction. The malware families with fewer than nine samples are removed to ensure reasonable splitting of dataset into training, validation, and test sets. Finally, 2071 samples are available, and the size of the data was approximately 30 GB. Training sets are used for learning, which involved fitting the parameters (i.e., weights) of a classifier. Validation sets are used to tune the parameters (i.e., architecture, not weights) of a classifier; for example, to choose the number of hidden units in a neural network. Test sets are used only to assess the performance (generalization) of a fully specified classifier. In our experiments, the ten-fold cross validation is adopted to train and test the model.

To evaluate the performance of 347-dim network traffic features and BIR-CNN model, the experimental results are compared with references^23,25,28–30^, which used different feature extractions and ML models. Based on the 347-dim network traffic features, to further evaluate the performance of the BIR-CNN model, the experimental results of BIR-CNN are compared with the results of the traditional ML model, namely (SVM—Support Vector Machine, DT—Decision Tree and RF—Random Forest), and with the traditional CNN model without batch normalization and inception-residual.

The BIR-CNN model consists of convolution layers, batch normalization, and inception-residual and shortcut connection modules. The kernel size is $3 \times 3,$ and the number of out channels in the four panels are 32, 64, 128, and 32; batch normalization parameters are set as 32, 64, 128, and 32. Inception and shortcut connection modules require $\mathrm{F}(\mathrm{x}) +\mathrm{ x}$; thus, the parameters are set the same as those for the upper layer. Subsequently, the fully connected layer is classified, where the dropout is set with a random deactivation probability of 0.5, and GAF is used as the activation function. The learning rate of 2-classifier is 0.001 and the L2 regularization term is 1.3e-2. The learning rate of 4-classifier is 0.00022 and the L2 regularization term of 2.588e-3. The learning rate of 35-classifier is 0.001 and the L2 regularization term is 0. Meanwhile, 2-, 4-classifier batches are 128 and 35-classifier batch is 256. The detailed parameters of BIR-CNN model are shown in Table 2.

**Table 2:** BIR-CNN model parameters.

Layers	Type	Kernel size/out_channel
L1		Batch Normalization (1)
L2	Conv	3 X 3/32
L3		Batch Normalization (32)
L4		Inception + Shortcut connection (32)
L5		Inception + Shortcut connection (32)
L6	Conv	3 X 3/64
L7		Batch Normalization (64)
L8		Inception + Shortcut connection (64)
L9		Inception + Shortcut connection (64)
L10	Conv	3 X 3/128
L11		Batch Normalization (128)
L12		Inception + Shortcut connection (128)
L13		Inception + Shortcut connection (128)
L14		Inception + Shortcut connection (128)
L15	Conv	3 X 3/32
L16		Batch Normalization (32)
L17		Inception + Shortcut connection (32)
L18		Inception + Shortcut connection (32)
L19		Dropout (0.5)
L20		Linear (3744, 1024)
L21		Dropout (0.5)
L22		Linear (1024,512)
L23		Dropout (0.5)
L24		Linear (512, 256)
L25		Linear (256, output_dim)

### Data cleanup

Prior to data training, the dataset is cleaned using the normal distribution triple principle to eliminate outliers. The SMOTE algorithm, which is a synthetic minority oversampling technique, is then used to solve the problem of uneven data distribution.

The 3*σ* principles of the normal distribution are as follows:

17 $$P\left( {\mu - 3\sigma } \right) < X \le \left( {\mu - 3\sigma } \right) = 99.7\% .$$

#### SMOTE algorithm

A small number of category samples are analyzed and simulated, and new manually simulated samples are added to the dataset, thus making the categories in the original data no longer severely imbalanced. The simulation process of this algorithm uses the KNN technique, and the steps to simulate the generation of new samples are as follows: (1) Sampling nearest neighbor algorithm to calculate *K* nearest neighbors for each minority class sample. (2) Randomly select N samples from the KNN algorithm for random linear interpolation. (3) Construct new minority class samples. (4) Synthesize the new samples with the original data to generate a new training set.

### Experimental results

In this part of the experiments, we compare the performance of the 347-dim network traffic features and BIR-CNN model with other state-of-the-art methods in the literature. It is worth mentioning that these methods used the CICAndMal2017 dataset. These 2-, 4-, 35-classifier results are provided in Tables 3, 4 and 5. Reference^23^ developed and extracted more than 80 network traffic features to detect and classify the malware. Reference^24^ extracted conversation-level network traffic features from the dataset can enhance the detection, categorization, and family classification of Android malware. Reference^26^ improved their malware category and family classification performance by combining the previous dynamic features (80 network-flows) with 2-g sequential relations of API calls. In reference^27^, the raw traffic is directly regarded as data input, so that the convolution neural network model automatically learns traffic features and performs classification. In reference^28^, 8115 features of the permissions and intent actions were acquired and saved in a CVS file. Then, the traffic network images feature data are produced which is introduced in methodology and saved as TFRecord files. The studies in^23,24,26^ used RF model and in^27,28^ used deep learning methods for Android malware classification. As shown in Table 3, BIR-CNN model achieve the highest accuracy of 0.99 and precision of 0.99 in malware binary classification. This deep learning model with network traffic features shows a much better performance than RF or other deep learning methods with improvements. In Table 4, BIR-CNN achieves a precision of 0.99 for malware 4-classification. Other studies^23,24,26,28^ achieved the precision of 0.50, 0.80, 0.83, 0.98, respectively. In Table 5, BIR-CNN model achieves a precision of 0.97 for malware 35-classification. Other studies^23,26,28^ achieved the precision of 0.28, 0.60, 0.73, respectively. obviously, the 35-classifier results are significantly improved by BIR-CNN model and 347-dim network traffic features.

**Table 3:** Performances of the proposed methods in 2-classifier.

Prediction method	Algorithm	Accuracy	Precision	Recall	F1-Score
Lashkari^23^	RF	–	0.86	0.88	–
Abuthawabeh^24^	RF	0.86	0.87	0.89	–
Taheri^26^	RF	–	0.95	0.95	–
Guo^27^	CNN	0.94	0.91	0.95	–
Feng^28^	CACNN	0.99	0.99	0.98	–
Our work	BIR-CNN	**0.99**	**0.99**	**0.99**	**0.99**

Significant values are in [bold].

**Table 4:** Performances of the proposed methods in 4-classifier.

Prediction method	Algorithm	Accuracy	Precision	Recall	F1-Score
Lashkari^23^	RF	–	0.50	0.49	–
Abuthawabeh^24^	RF	0.80	0.80	0.80	–
Taheri^26^	RF	–	0.83	0.81	–
Feng^28^	CACNN	0.98	0.98	0.96	–
Our work	BIR-CNN	**0.99**	**0.98**	**0.99**	**0.99**

Significant values are in [bold].

**Table 5:** Performances of the proposed methods in 35-classifier.

Prediction method	Algorithm	Accuracy	Precision	Recall	F1-Score
Lashkari^23^	RF	–	0.28	0.26	–
Taheri^26^	RF	–	0.60	0.61	–
Feng^28^	CACNN	0.70	0.73	0.74	–
Our work	BIR-CNN	**0.95**	**0.97**	**0.95**	**0.95**

Significant values are in [bold].

Based on the 347-dim network traffic features, the performance of BIR-CNN is compared with DT, RF, SVM and CNN for 2-, 4-, 35- classifier. The higher the values of accuracy, precision, recall, and F1 score, the better the performance of the model. Table 6 shows the 2-classifier performance of each ML model on the test dataset. The results reveal that the performance of the DL model is superior to that of the traditional ML models. In addition, the overall performance of the BIR-CNN model is better than that of the traditional CNN model. The BIR-CNN model performed best in the four evaluation indexes. For example, the recall value of the traditional ML methods and CNN are not high; although SVM can achieve a recall value of 0.89, the recall value of the BIR-CNN model proposed in this paper is 0.99.

**Table 6:** Performances of five models in 2-classifier.

Model	Accuracy	Precision	Recall	F1-score
DT	0.91	0.91	0.91	0.91
RF	0.92	0.92	0.91	0.92
SVM	0.89	0.91	0.90	0.89
CNN	0.94	0.93	0.93	0.94
BIR-CNN	**0.99**	**0.99**	**0.99**	**0.99**

Significant values are in [bold].

Table 7 presents the 4-classifier performances of the BIR-CNN, CNN, SVM, DT, RF models respectively on each category of malicious software. Averaging the results for the four categories, the BIR-CNN model achieves the best recall (0.99) and F1-score (0.99). On the contrary, for SVM, the recall value is 0.86 and the F1-score is 0.85; for RF, the recall and F1-score are 0.87. Overall, the BIR-CNN model outperforms the other three models. Neural networks, especially CNN, are increasingly being used in malware detection and classification due to their advantages in processing raw data and their ability to learn features.

**Table 7:** Performances of five models in 4-classifier.

Model	Accuracy	Precision	Recall	F1-score
DT	0.85	0.85	0.85	0.85
RF	0.87	0.87	0.87	0.87
SVM	0.90	0.86	0.86	0.85
CNN	0.91	0.87	0.89	0.87
BIR-CNN	**0.99**	**0.98**	**0.99**	**0.99**

Significant values are in [bold].

Table 8 presents the 35-classifier results. The average values of the BIR-CNN model are much higher than those of other models in the four evaluation indexes, which are almost 1.00. The average recall of DT is only 0.81, which is 0.18 less than that of the BIR-CNN model, and its average precision rate is 0.81, which is also 0.18 less than that of BIR-CNN. The average accuracy, precision, recall, and F1-score are around 0.84 of RF, SVM and CNN. These evaluation criteria reveal that the 347-dim network traffic features and BIR-CNN model proposed in this paper has a markedly superior performance for multi classification.

**Table 8:** Performances of five models in 35-classifier.

Model	Accuracy	Precision	Recall	F1-score
DT	0.79	0.81	0.81	0.79
RF	0.81	0.84	0.83	0.82
SVM	0.86	0.84	0.81	0.81
CNN	0.87	0.84	0.81	0.81
BIR-CNN	**0.99**	**0.99**	**0.99**	**0.99**

Significant values are in [bold].

**Figure 4:**
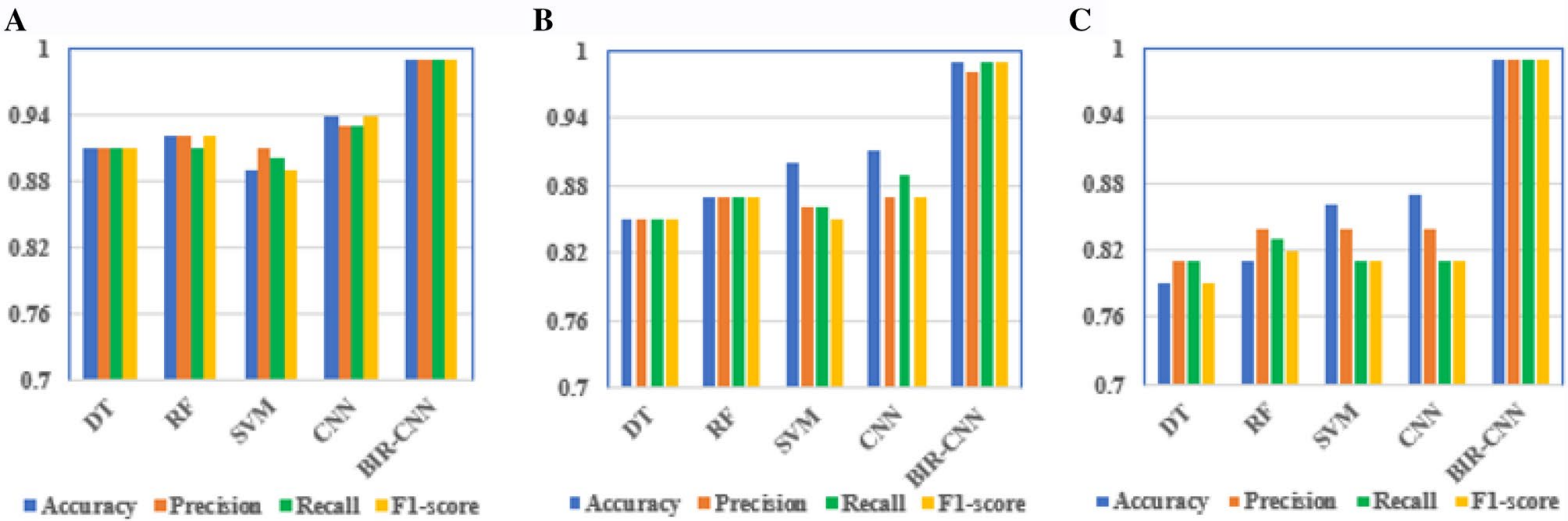
The ten-fold cross validation results of DT, RF, SVM, CNN, BIR-CNN models. (**A**) Performances of five models in 2-classifier. (**B**) Performances of five models in 4-classifier. (**C**) Performances of five models in 35-classifier.

The result distributions of the DT, RF, SVM, CNN, and BIR-CNN models reflect their performance more intuitively, which are illustrated in Fig. 4.

**Figure 5:**
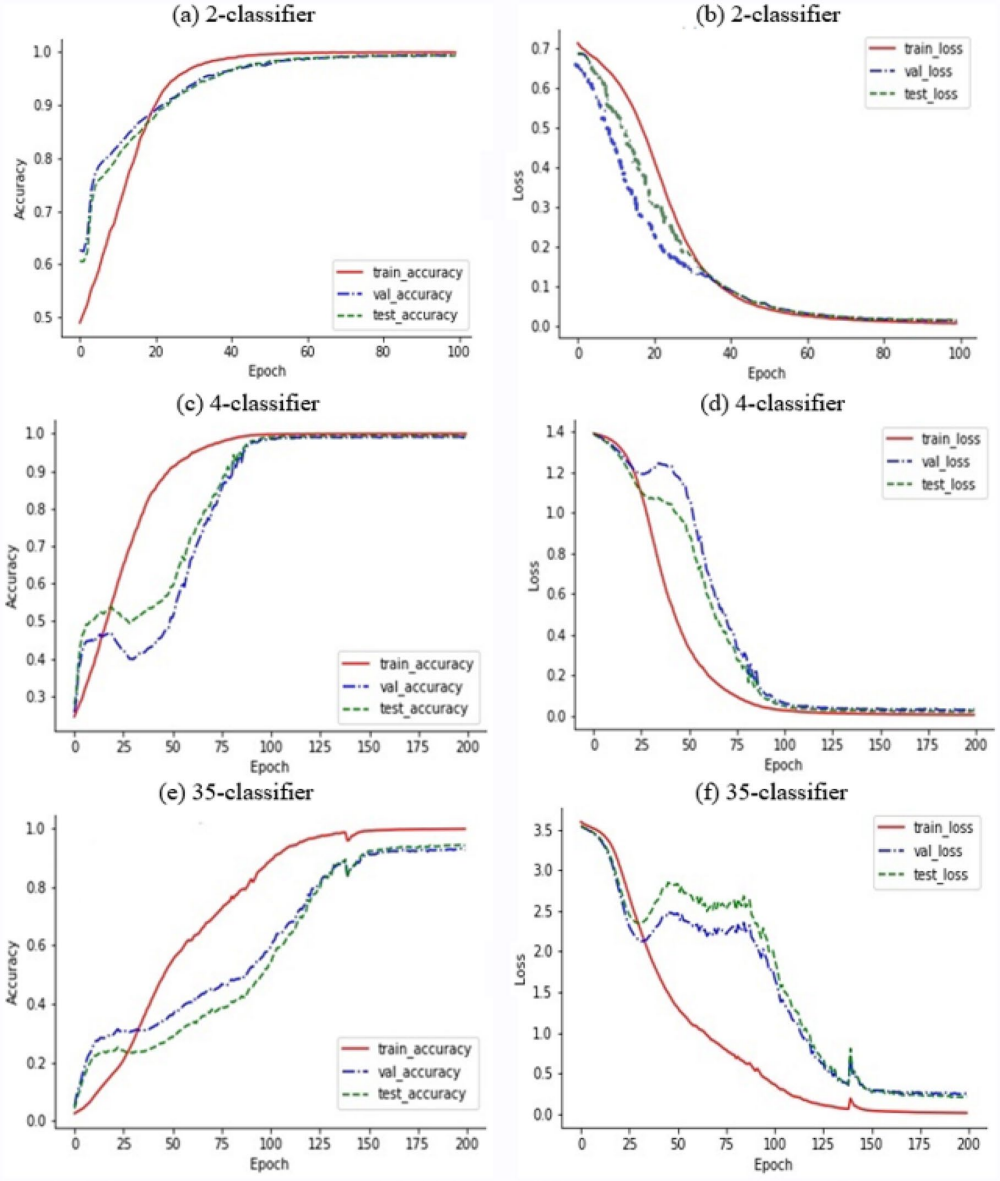
Accuracy and loss curves of the BIR-CNN model. (**a**) and (**b**) are accuracy and loss curves of 2-classifier respectively. (**c**) and (**d**) are accuracy and loss curves of 4-classifier respectively. (**e**) and (**f**) are accuracy and loss curves of 35-classifier respectively.

To show the performance of the BIR-CNN model in malware classification in a more intuitive manner, Fig. 5 illustrates the accuracy curves and loss curves of BIR-CNN on the training, testing, and validation dataset. BIR-CNN achieves an impressive accuracy of 99.96%, 99.49%, and 99.34% in training samples, validation samples, and testing samples, respectively, in binary classification (2-classifier) (Fig. 5a); 99.98%, 98.95%, and 99.37% in training samples, validation samples, and testing samples, respectively, in category classification (4-classifier) (Fig. 5c); and 99.70%, 92.52%, and 94.02% in training samples, validation samples, and testing samples, respectively, in malicious family classification (35-classifier) (Fig. 5e). These results show that BIR-CNN performs well in 2-, 4-, and 35-classifiers. The loss in binary classification (2-classifier) is from 0.712427–0.008237 in the training sample, 0.685336–0.014405 in the validation sample, and 0.686690–0.015525 in the testing sample (Fig. 5b). The loss in category classification (4-classifier) is 1.389648–0.004504 in the training sample, 1.386036–0.031262 in the validation sample, and 1.384183–0.020317 in the test sample (Fig. 5d). The loss in malicious family classification (35-classifier) is 3.594075–0.013753 in the training sample, 3.541312–0.256138 in the validation sample, and 3.543989–0.215694 in the test sample (Fig. 5f). It can be noticed from Fig. 4 that the 35-classifier performs smoothly after 150 cycles.

**Figure 6:**
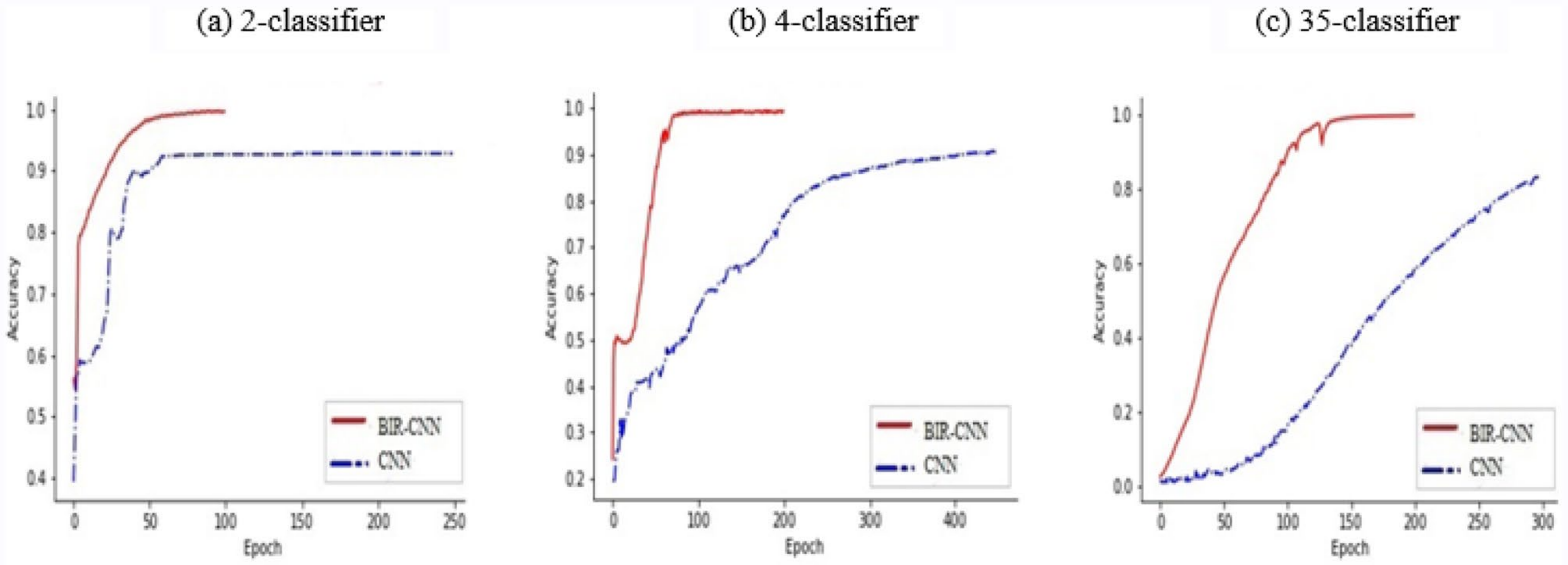
Accuracy curves of BIR-CNN and CNN for comparison for (**a**) 2-classifier, (**b**) 4-classifier, (**c**) 35-classifier, respectively.

Figure 6 shows a comparison of CNN and BIR-CNN in binary classification (2-classifier), category classification (4-classifier), and malicious family classification (35-classifier). There is a clear difference between the accuracy curves of CNN and BIR-CNN in binary classification and multi-classification. In binary classification (2-classifier), the accuracy of BIR-CNN was 0.489729–0.999574, whereas that of CNN was only 0.940947 after 250 cycles. BIR-CNN achieved an accuracy of 0.999669, but CNN achieved a value of 0.910228 in 4-classifier. In malicious family classification (35-classifier), BIR-CNN converged after 200 cycles and was finally able to reach 0.997460, but the CNN converged slowly and was only able to reach 0.866390, indicating that the proposed model performs well in terms of accuracy in binary classification, and both classifiers perform smoothly after 200 cycles.

**Figure 7:**
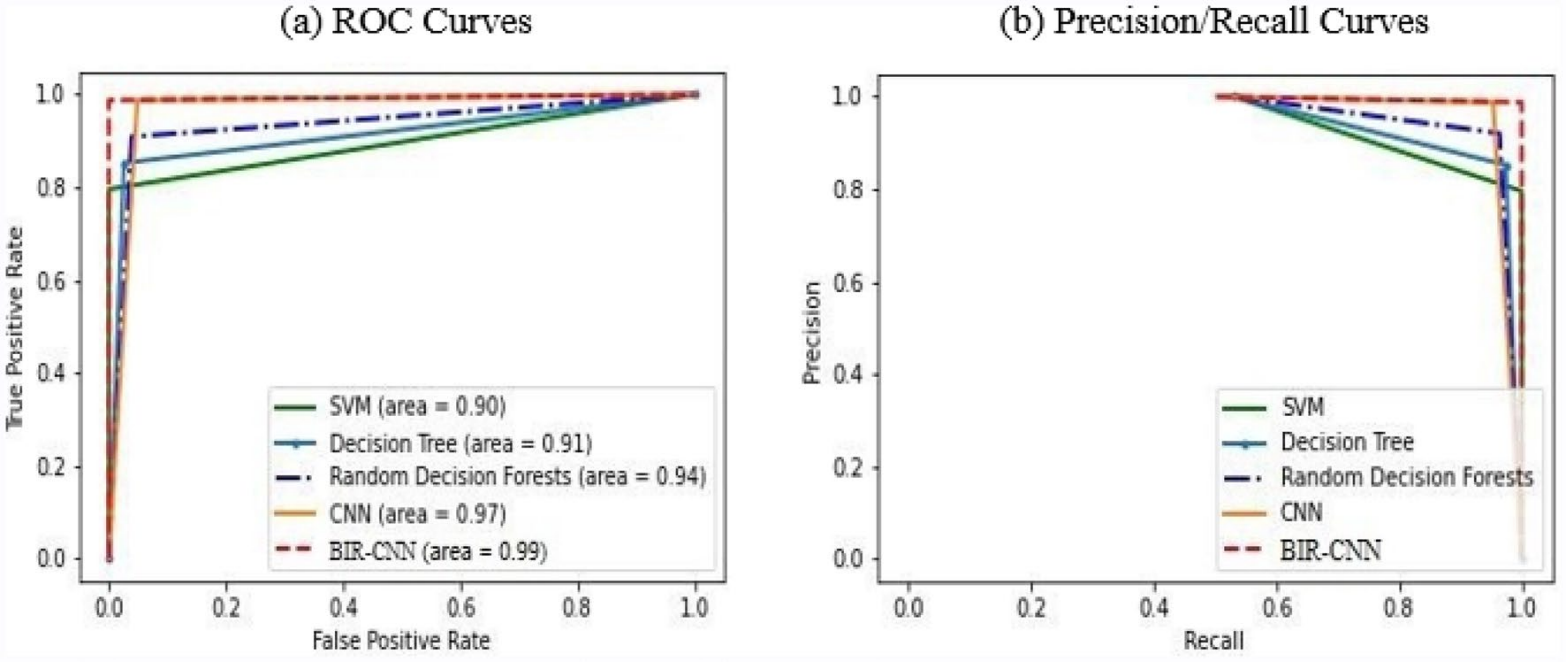
ROC curves (**a**) and PR curves (**b**) for the five models to classification malicious software.

The ROC curves and PR curves are illustrated in Fig. 6 to show the advantage of BIR-CNN. Figure 7a shows the ROC curves for the five models. AUC refers to the area under the ROC curve. The larger the AUC, the more effective the classifier will be. The AUC value of BIR-CNN is 0.99, which is significantly greater than that of SVM, DT, RF, and CNN. Compared to the AUC value of SVM is 0.91, the AUC value of BIR-CNN is 0.99. Therefore, BIR-CNN will become a useful tool in the classification of malicious software or at least complementary to existing methods. The PR curves of five ML models are shown in Fig. 7b which illustrate the relationship between precision and recall. The graph of precision and recall curve is used to compare the classification performance. When the gap between positive and negative samples is not large, the trend of the ROC curve and PR curve are the same; however, when there are many negative samples, the two differ greatly. The ROC effect still seems to be very good, but the PR is the reflection of the general effect. From Fig. 7, we can conclude that BIR-CNN model exhibits the best performance.

**Figure 8:**
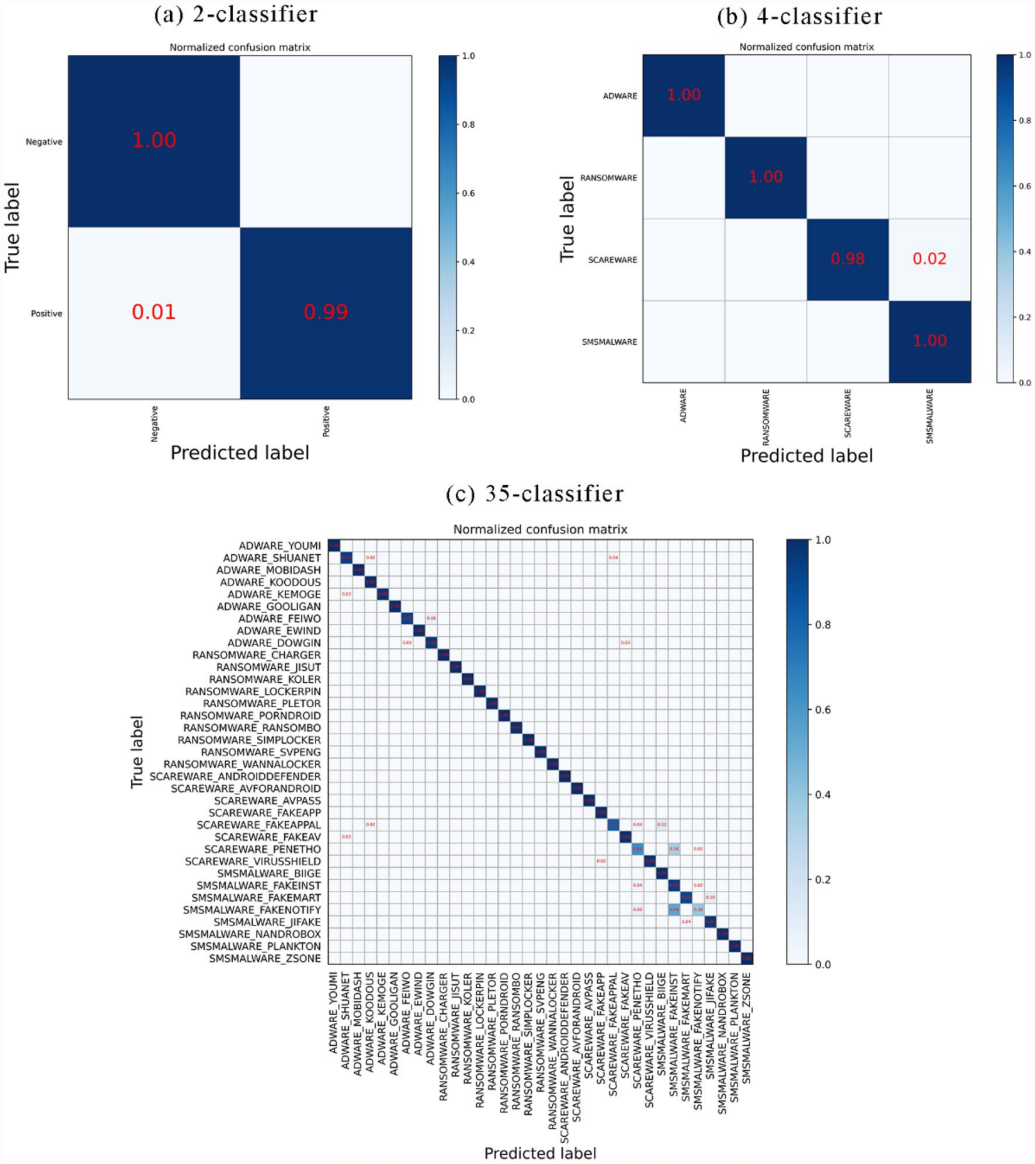
Confusion matrix for (**a**) 2-classifier, (**b**) 4-classifier, (**c**) 35-classifier of BIR-CNN model.

The confusion matrix values are composed of the TP and FN rates of the malicious code classification. The abscissa in the confusion matrix represents the neural network prediction classification. The ordinate represents the true classification, and the numbers on the diagonal denote the number of correct classifications by the neural network. The numbers outside the diagonal denote the number of inconsistencies between the predicted and actual classifications, indicating the number of incorrect classifications by the neural network. Figure 8 shows the results of confusion matrix for malware 2-classifier (Fig. 8a), 4-classifier (Fig. 8b), and 35-classifier (Fig. 8c) respectively on the test data. From the confusion matrix results, it can be concluded that the BIR-CNN model performs well on the dataset. Based on the confusion matrix, we calculate the Kappa coefficient, which is used to measure the model classification effect. The Kappa coefficient values can be as high as 0.99 for malware detection and category classification, and 0.95 for 35 classification.

To further verify the performance of BIR-CNN model in detecting and classifying Android malware on other dataset, CCCS-CIC-AndMal-2020 dataset^33,34^, Canadian Institute for Cybersecurity (CIC) project in collaboration with Canadian Centre for Cyber Security (CCCS), is conducted. The dataset includes 200 K benign and 200 K malware samples totaling to 400 K android apps with 14 prominent malware categories and 191 eminent malware families. Benign android apps (200 K) are collected from Androzoo dataset to balance the huge dataset. The 14 malware categories are collected including Adware, Backdoor, File Infector, No Category, Potentially Unwanted Apps (PUA), Ransomware, Riskware, Scareware, Trojan, Trojan-Banker, Trojan-Dropper, Trojan-SMS, Trojan-Spy and Zero-day. Table 9 presents the details of 14 android malware categories along with number of respective families and samples in the dataset. The extracted features include memory, API, network, battery, logcat, and process.

**Table 9:** The details of CCCS-CIC-AndMal-2020 dataset.

Category	Number of families	Number of samples	Category	Number of families	Number of samples
Adware		47,210	Scareware	3	1556
Backdoor	11	1,538	Trojan	45	13,559
File Infector	5	669	Trojan-Banker	11	887
No Category	–	2,296	Trojan-Dropper	9	2302
PUA	8	2,051	Trojan-SMS	11	3125
Ransomware	8	6,202	Trojan-Spy	11	3540
Riskware	21	97,349	Zero-day	–	13,340

**Figure 9:**
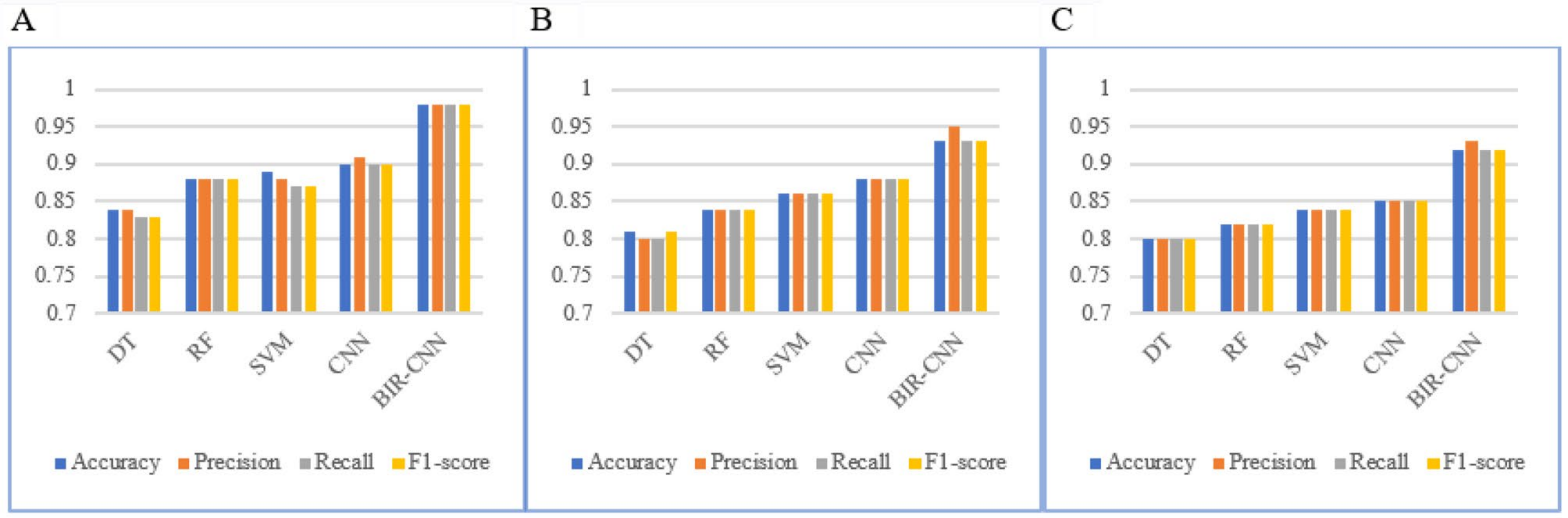
The ten-fold cross validation results of DT, RF, SVM, CNN, BIR-CNN models. (**A**) Performances of five models in 2-classifier. (**B**) Performances of five models in 14-classifier. (**C**) Performances of five models in 191-classifier.

**Figure 10:**
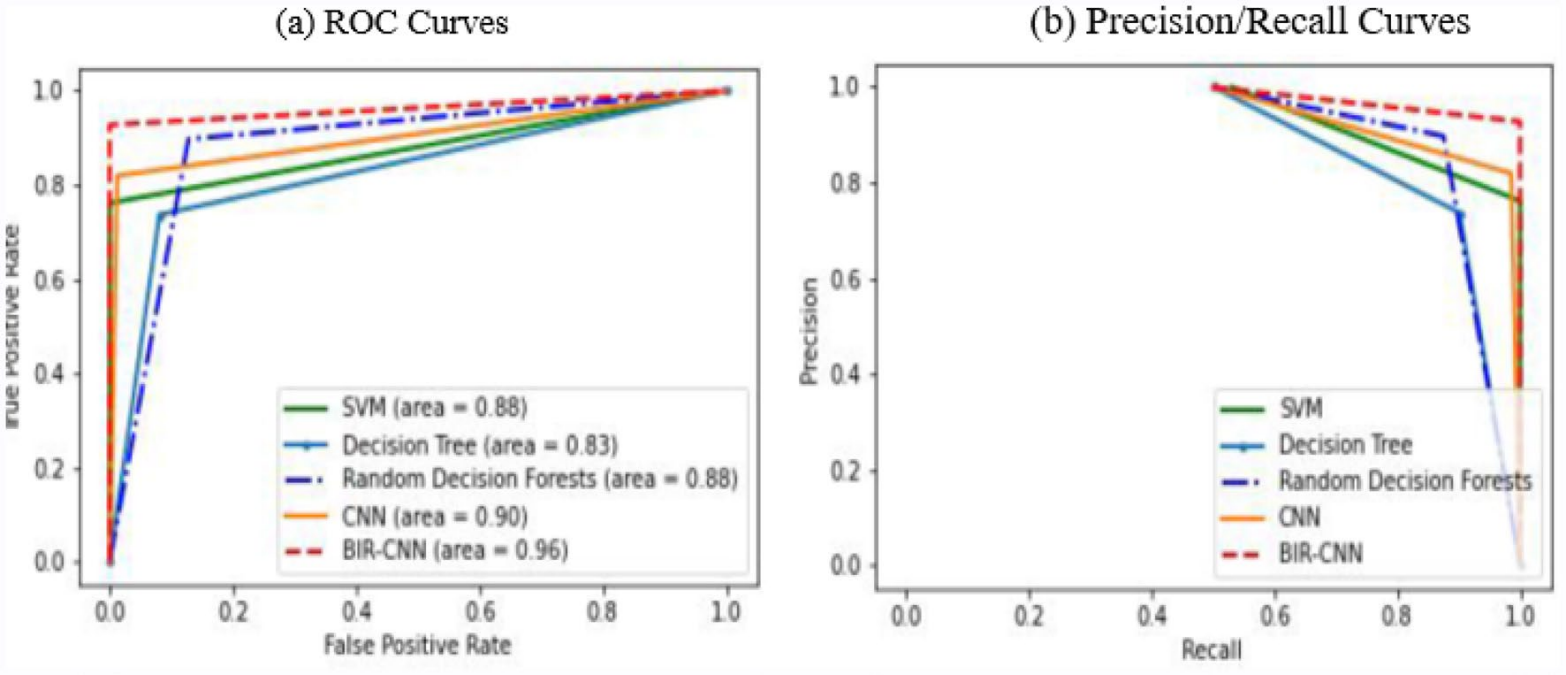
ROC curves (**a**) and PR curves (**b**) for the five models to classification malicious software.

**Figure 11:**
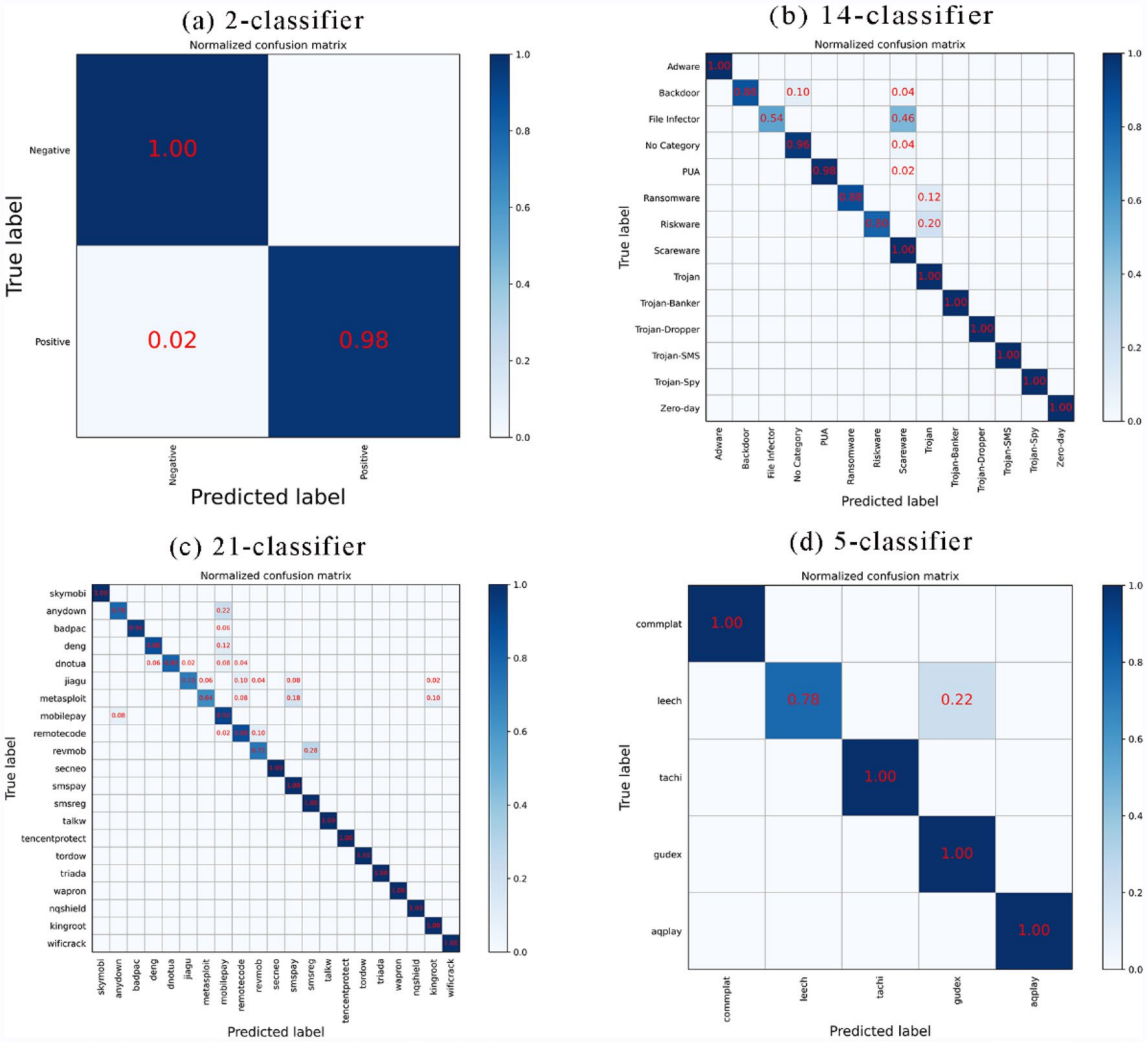
Confusion matrix of BIR-CNN model for (**a**) 2-classifier, (**b**) 14-classifier, (**c**) 21-classifier (families) of Riskware category, (**d**) 5-classifier (families) of File Infector category.

The experimental results are shown in the following Tables 10, 11 and 12 and Figs. 9, 10 and 11. Tables 10, 11 and 12 display the results of 2-classifier, 14-classifier and 191-classifier respectively of five machine learning models. Figures 9, 10 visually illustrate the comparison results of BIR-CNN model with other models respectively. Figure 11 are confusion matrix for 2-classifier, 14-classifier, 21-classifier (families) of Riskware category and 5-classifier (families) of File Infector category. The Riskware category has 21 families and obtains the most samples in 191 families. The File Infector category has 5 families and obtains the least samples. From all the results, the BIR-CNN model obtain the best results including accuracy, precision value, recall, F1-score, AUC. More significantly, the BIR-CNN model achieves good performance in multi classification.

**Table 10:** Performances of five models in 2-classifier.

Model	Accuracy	Precision	Recall	F1-score
DT	0.84	0.84	0.83	0.83
RF	0.88	0.88	0.88	0.88
SVM	0.89	0.88	0.87	0.87
CNN	0.90	0.91	0.90	0.90
BIR-CNN	**0.98**	**0.98**	**0.98**	**0.98**

Significant values are in [bold].

**Table 11:** Performances of five models in 14-classifier.

Model	Accuracy	Precision	Recall	F1-score
DT	0.81	0.80	0.80	0.81
RF	0.84	0.84	0.84	0.84
SVM	0.86	0.86	0.86	0.86
CNN	0.88	0.88	0.88	0.88
BIR-CNN	**0.93**	**0.95**	**0.93**	**0.93**

Significant values are in [bold].

**Table 12:** Performances of five models in 191-classifier.

Model	Accuracy	Precision	Recall	F1-score
DT	0.80	0.80	0.80	0.80
RF	0.82	0.82	0.82	0.82
SVM	0.84	0.84	0.84	0.84
CNN	0.85	0.85	0.85	0.85
BIR-CNN	**0.92**	**0.93**	**0.92**	**0.92**

Significant values are in [bold].

## Discussion and conclusion

This paper presented an Android malware classification method (BIR-CNN) based on 347-dim network traffic features. The classification performance of the proposed model was determined and compared with those of other models by using the CICAndMal2017 dataset. Furthermore, ten-fold cross validation was used for deep learning model selection and evaluation. Cross validation, sometimes called rotation estimation, is a statistically practical method to cut samples into smaller subsets. The purpose of cross validation is to make the evaluated model more accurate and reliable. In order to obtain a reliable and stable model, cross validation is used to evaluate the prediction performance of the model, especially the performance of the trained model on new data. To some extent, over-fitting can be reduced by cross validation. Experimental results in Tables 3, 4, 5, 6, 7 and 8 shown that, the values of classification accuracy, precision, recall, and F1-score of BIR-CNN for most malware were 1.00 compared with those of traditional ML methods (SVM, DT, and RF). The other dataset CCCS-CIC-AndMal-2020 was tested to verity the classification performance of the BIR-CNN and obtained the best results.

The inception-residual network modules are efficient and fast in processing network traffic data because of their ability to accelerate data computation, deepen the network, and increase the non-linearity of the network. Moreover, regularization can speed up the convergence of the deep model training process. Thus, the BIR-CNN model proposed in this paper is effective for the classification of Android malware.

Taken together, we can also draw the following further research directions: One of our future research directions is to use this DL model BIR-CNN for the classification of Android new emerging software, whether they are benign or malicious, and further determine their category and family. Another direction is to process different network traffic dataset, extract their static and dynamic features, and input them into the BIR-CNN model; this proves the generalizability of the proposed BIR-CNN model.

## Data Availability

The dataset used and/or analysed during the current study available from the corresponding author on reasonable request.
